# Human versus Rat PRF on Collagen Membranes: A Pilot Study of Mineralization in Rat Calvaria Defect Model

**DOI:** 10.3390/bioengineering11050414

**Published:** 2024-04-23

**Authors:** Karol Ali Apaza Alccayhuaman, Patrick Heimel, Stefan Tangl, Stefan Lettner, Carina Kampleitner, Layla Panahipour, Ulrike Kuchler, Reinhard Gruber

**Affiliations:** 1 Department of Oral Biology, University Clinic of Dentistry, Medical University of Vienna, 1090 Vienna, Austria; caroline7_k@hotmail.com (K.A.A.A.); layla.panahipour@meduniwien.ac.at (L.P.); 2 Karl Donath Laboratory for Hard Tissue and Biomaterial Research, University Clinic of Dentistry, Medical University of Vienna, 1090 Vienna, Austria; patrick.heimel@trauma.lbg.ac.at (P.H.); stefan.tangl@meduniwien.ac.at (S.T.); stefan.lettner@meduniwien.ac.at (S.L.); carina.kampleitner@meduniwien.ac.at (C.K.); 3 Austrian Cluster for Tissue Regeneration, 1200 Vienna, Austria; 4 Ludwig Boltzmann Institute for Traumatology, The Research Center in Cooperation with AUVA, 1200 Vienna, Austria; 5 Department of Oral Surgery, University Clinic of Dentistry, Medical University of Vienna, 1090 Vienna, Austria; ulrike.kuchler@meduniwien.ac.at; 6 Department of Periodontology, School of Dental Medicine, University of Bern, 3010 Bern, Switzerland

**Keywords:** PRF, homologous, rat, calvaria defect, bone regeneration, collagen membranes, heterologous

## Abstract

Platelet-rich fibrin, the coagulated plasma fraction of blood, is commonly used to support natural healing in clinical applications. The rat calvaria defect is a standardized model to study bone regeneration. It remains, however, unclear if the rat calvaria defect is appropriate to investigate the impact of human PRF (Platelet-Rich Fibrin) on bone regeneration. To this end, we soaked Bio-Gide^®^ collagen membranes in human or rat liquid concentrated PRF before placing them onto 5 mm calvarial defects in Sprague Dawley rats. Three weeks later, histology and micro-computed tomography (μCT) were performed. We observed that the collagen membranes soaked with rat PRF show the characteristic features of new bone and areas of mineralized collagen matrix, indicated by a median mineralized volume of 1.5 mm^3^ (range: 0.9; 5.3 mm^3^). Histology revealed new bone growing underneath the membrane and hybrid bone where collagen fibers are embedded in the new bone. Moreover, areas of passive mineralization were observed. The collagen membranes soaked with human PRF, however, were devoid of histological features of new bone formation in the center of the defect; only occasionally, new bone formed at the defect margins. Human PRF (h-PRF) caused a median bone volume of 0.9 mm^3^ (range: 0.3–3.3 mm^3^), which was significantly lower than what was observed with rat PRF (r-PRF), with a BV median of 1.2 mm^3^ (range: 0.3–5.9 mm^3^). Our findings indicate that the rat calvaria defect model is suitable for assessing the effects of rat PRF on bone formation, but caution is warranted when extrapolating conclusions regarding the efficacy of human PRF.

## 1. Introduction

Platelet-rich fibrin is prepared from fractionated blood, where the plasma components are separated from the erythrocytes before coagulation [1,2]. The plasma fraction containing the platelets and leukocytes undergoes coagulation, and the resulting yellow clot is removed while serum is squeezed out, ending up with PRF membranes [1]. PRF membranes have a broad spectrum of clinical applications ranging from dentistry [3,4,5,6,7,8,9] to other disciplines, such as healing diabetic ulcers [10], cartilage and joint repair [11], and even pterygium surgery [12]. In dentistry, for instance, a typical application of PRF membranes is in filling extraction sockets, not only for alveolar ridge preservation but also for the improvement of bone regeneration [13,14]. Moreover, using liquid PRF to prepare a conglomerate of PRF membranes with a graft, named sticky bone, is widely established [15,16]. Besides the application of PRF membranes, PRF is used to coat and functionalize surfaces and implants [17,18,19]. Even though PRF has become a clinically established treatment option, it requires preclinical models to unravel the underlying cellular and molecular mechanisms, as in vitro models are meaningful but have limitations [20]. Preclinical rat calvaria defect models revealed the impact of various preparations of rat PRF; however, bone regeneration was restricted to the defect margin [21,22]. PRF was also combined with a bone substitute material, allowing for bridging of the defects in the rat calvaria defect model [23,24]. Thus, PRF cannot replace a scaffold guiding the new bone towards the center of the defect, but collagen membranes can [25,26,27,28,29,30,31]. Preclinical research revealed that collagen-based scaffolds were functionalized with platelet-rich plasma for enhanced skin wound healing [32]. Moreover, bilayer collagen membranes reinforced with beta-tricalcium-phosphate particles in combination with rabbit PRF were used to evaluate bone regeneration in rabbits in vivo [33]. Among all the strategies introduced, the most commonly and clinically implemented concept is using PRF for the preparation of sticky bone, where bone grafts are combined with pieces of solid PRF and mixed up with liquid PRF. Coagulation allows the formation of a conglomerate of fibrin-rich matrix where all the ingredients are entrapped, allowing for good handling of the graft; presumably, the PRF components support the process of graft consolidation, which is essentially bone regeneration. Some preclinical studies have investigated wound healing using human platelet-rich fibrin (PRF) to assess its effects [34,35]. The rationale for using human donors stems from limitations such as the lower volume of PRF and faster coagulation compared to animal models [36]. Thus, there is a demand for preclinical models to study how PRF prepared from human blood affects the properties of biomaterials such as collagen membranes used to treat defects.

Bio-Gide^®^ collagen membranes have a long history of guided bone regeneration [37] and have recently been reported to possess osteoconductive properties, at least in a rat calvaria defect model [25,26,27,28,29,30,31]. Histology revealed new bone formation and areas of mineralized matrix inside the spongy part of the collagen membrane, and this model was appropriate to study the impact of acid bone [27] and dentine lysate [26] as well as bone-conditioned media [28] on bone regeneration. This collagen membrane model is presumably also appropriate to study the impact of rat PRF on bone regeneration, mainly because similar attempts have been clinically made. However, it remains unclear whether the rat calvaria defect model covered with a functionalized collagen membrane is suitable for studying human PRF. This uncertainty arises from the lack of established preclinical models optimized for human PRF, with most in vitro protocols being tailored to human PRF [20]. Additionally, previous preclinical studies have primarily utilized heterologous PRF resources, with limited investigations into homologous PRF resources. Thus, the aim of this study is to compare the effects of human and rat PRF on the osteoconductive properties of Bio-Gide^®^ collagen membranes. Our findings suggest that while rat PRF promotes new bone formation within the collagen membranes, human PRF functionalization attenuates this process, indicating that the rat calvaria defect model may not be ideal for studying human PRF effects.

## 2. Material and Methods

### 2.1. Study Design

**Figure 1:**
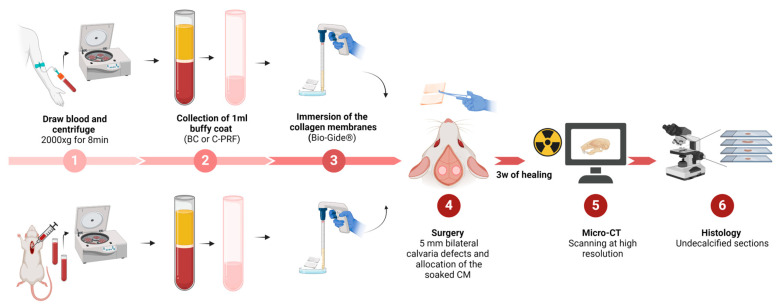
Visualization of the systematic steps outlining the study design workflow for the current research. BC refers to buffy coat, which is similar to what we consider concentrated PRF (C-PRF).

The experiment was conducted at the Department of Biomedical Research, following the ethical guidelines of the ARRIVE (Animal Research: Reporting of In Vivo Experiments) framework. The authorization for animal research was obtained from the ethical review board of the host institution, the Medical University of Vienna, and clearance was also obtained from the Austrian Federal Ministry of Education, Science, and Research (Approval No. BMWFW-66.009/0217-WF/V/3b/2017). Ten adult male Sprague Dawley rats weighing 200–300 g were randomly selected for our study. These rats were categorized into cohorts, each receiving collagen membranes (Bio-Gide^®^, Geistlich, Wolhusen, Switzerland) soaked with either human or rat concentrated PRF (C-PRF) for at least 10 min [38,39,40]. The animals were housed in a controlled environment, provided ad libitum access to food and water, and subjected to a 12 h light/dark cycle regimen. The assignment of treatments remained undisclosed to the operating surgeon until the moment of membrane placement was necessitated for the defect. Throughout the analytical phase, the assessment examiners were blinded to the treatment allocations (Figure 1).

### 2.2. Preparation of C-PRF

To obtain liquid C-PRF [38,39,40], volunteers signed an informed consent, and the ethics committee of the Medical University of Vienna (1644/2018) approved the preparation of PRF. Venous blood samples were acquired from two healthy volunteers, 23 and 35 years old, using plastic tubes without additives (‘No Additive’ tubes, Greiner Bio-One GmbH, Kremsmünster, Austria). Human blood was centrifuged at 2000× *g* for 8 min utilizing a swing-out rotor (Z306 Hermle, Universal Centrifuge, Wehingen, Germany). The 1 mL buffy coat (BC or C-PRF; [38,39,40]) was collected to immerse the membranes. Rat C-PRF was prepared according to the same protocol following blood collection via cardiac puncture using a 10 mL syringe. Membranes were immersed in C-PRF prepared from rat blood in the same way as for the human C-PRF. Again, immersion time was at least 10 min, allowing the C-PRF to coagulate.

### 2.3. Surgery

Anesthesia was induced in the rats for the surgical procedure, employing an intraperitoneal injection of medetomidine (0.15 mg/kg), midazolam (2 mg/kg) and fentanyl (5 µg/kg). Using a trephine drill with a 5 mm outer diameter, bilateral 5 mm defects were created in the parietal bone. Following random treatment assignments, defects were covered with a collagen membrane containing either human or rat PRF, blinded to the surgeon. A 6 × 6 mm collagen membrane was inserted for each group, ensuring at least a 1 mm overlap with the defect’s perimeter at all points. Wound closure was performed using a two-layer technique with absorbable USP 5-0 sutures and atipamezole (0.75 mg/kg) and flumazenil (0.2 mg/kg) were injected subcutaneously to reverse the sedative effect. Postoperative pain was mitigated by administering piritramide orally by adding 30 mg piritramide and 10 mL 10% glucose solution to 250 mL drinking water. To conclude the procedure, after a three-week healing period, rats were euthanized through an intracardial overdose of pentobarbital.

### 2.4. Micro-CT Analysis

Tissue samples were fixed using phosphate-buffered formalin (Roti-Histofix 4%, Carl Roth, Karlsruhe, Germany). The micro-CT scans were performed at 90 kV and 200 A, with an isotropic resolution of 10.3 µm and a 500 ms integration time (Scanco Medical AG, Bruttisellen, Switzerland). Following the scans, an open-source image processing program (FIJI, ImageJ, National Institutes of Health, Bethesda, MD, USA) was employed to adjust the image orientation, aligning the drill direction along the Z-axis and positioning the defect near the image center [41]. The Region of Interest (ROI) was manually delineated, and using a circular cylinder aligned with the center of the defect, automatic segmentation was achieved by setting a threshold of 350 mgHA/cm^3^ to differentiate mineralized tissue from the background. An ImageJ ruleset was developed to streamline the process, enabling automatic ROI segmentation from CT images. Subsequently, various parameters, including bone volume (BV) and defect coverage (Cov%), were quantified.

### 2.5. Histological Analysis

The tissue specimens underwent a progressive dehydration process using increasing alcohol concentrations and were subsequently embedded in a light-curing resin (Technovit 7200 VLC + BPO; Kulzer & Co., Wehrheim, Germany). Precise thin-ground sections were then meticulously prepared, following the sagittal suture and centered on the defect, employing visualization software (Amira-Avizo 3D 2021.2, Thermo Fisher Scientific, Waltham, MA, USA). Further processing of the resin blocks involved cutting and grinding equipment (Exakt Apparatebau, Norderstedt, Germany). The prepared sections were stained using Levai-Laczko dye, combining azure II, methylene blue and pararosaniline. Systematic scanning and evaluation of the stained slides were conducted using an Olympus BX61VS digital virtual microscopy system (DotSlide 2.4; Olympus, Japan, Tokyo), equipped with a 20X objective providing a resolution of 0.32 µm per pixel. A comprehensive descriptive analysis was performed on the microscopy data to capture additional intricacies and details meticulously.

### 2.6. Statistics

The micro-CT data were analyzed using descriptive statistics. The parameters examined in this study were bone volume (BV) and defect coverage (Cov%) between the defects that were treated with a collagen membrane soaked in human PRF and those that were treated with rat PRF. The Mann–Whitney U test was applied for statistical analysis. The statistical calculations were performed using Prism v7 (GraphPad Software, La Jolla, CA, USA), and R version 4.0.2 was used for analyses. The significance level was set at *p* < 0.05. The sample size calculation was performed using G*power 4 (Düsseldorf, Germany) based on data from a previous study [28]. Considering a bone volume of 0.85 mm^3^ ± 0.26 for the control group and 0.38 mm^3^ ± 0.46 in the treated group, with an 80% power and type I error rate of 5%, we estimated a sample size of 9 animals per group. Using ANOVA, a secondary analysis was performed among the h-PRF, r-PRF, and Empty Control. In cases of significance, we followed up with post hoc tests against the Empty Control. The related tables are in Supplementary Materials (Tables S1 and S2).

## 3. Results

### 3.1. Histological Analysis

**Figure 2:**
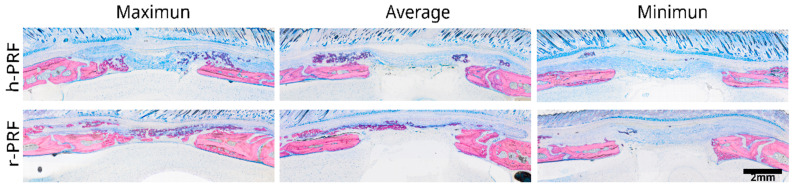
Representative images displaying the extent of new bone formation in the group treated with a collagen membrane soaked in human PRF (h-PRF) compared to the group treated with rat PRF (r-PRF). The images showcase the maximum, average, and minimum levels of new bone formation identified by µCT based on the histological samples.

Selected samples were prepared for histological analysis from both experimental groups, one treated with a collagen membrane soaked in human C-PRF and the other with a collagen membrane soaked in rat C-PRF (Figure 2).

### 3.2. Collagen Membrane Soaked in Human PRF (h-PRF)

**Figure 3:**
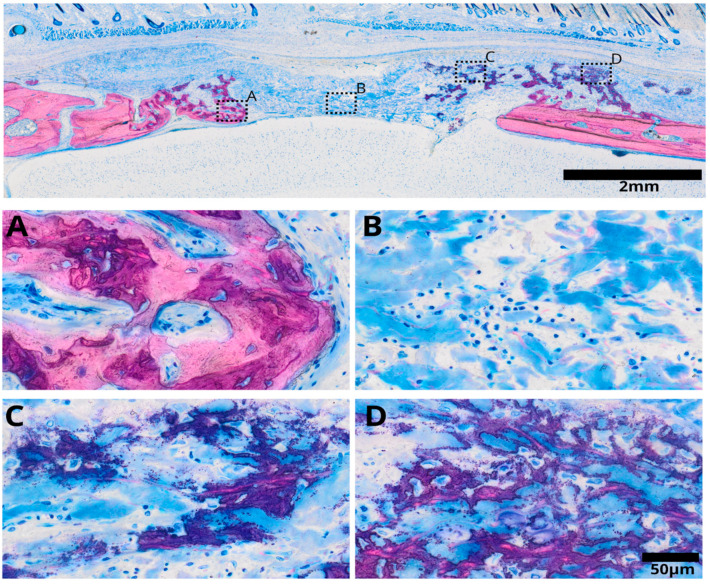
Overview image illustrating the collagen membrane immersed in human platelet-rich fibrin (h-PRF) after a three-week healing period, displaying distinct regions with varied characteristics. In the inner part of the defect (**A**), mature bone formation is evident, featuring osteon formation and a lamellar structure. The central zone of the defect (**B**) contains densely stained collagen fibers from the remaining collagen membrane. Additionally, the remaining collagen membrane shows a denser reticular structure, indicating an area of mineralized fibers integrated into the healing process (**C**). This characteristic fiber mineralization exhibits a reticular pattern, depicting a blend of bone structure intertwined with collagen fibers, visualized as dense blue staining (**D**).

After three weeks of healing, distinct differences were observed in various regions of the collagen membrane immersed in h-PRF (Figure 3). Adjacent to the defect’s edge, mature bone formation was evident, accompanied by the invasion of lamellar bone into the collagen membrane. Conversely, the central portion of the defect exhibited densely stained collagen fibers surrounded by inflammatory infiltrate. In areas proximal to the periosteal bone, a substantial presence of mineralized fibers was observed, giving rise to a hybrid bone appearance. This hybrid bone structure displayed a unique reticular formation entrapping cells, with evident mineralization enveloping these fibers, interspersed with collagen fibers from the membrane.

### 3.3. Collagen Membrane Soaked in Rat PRF (r-PRF)

**Figure 4:**
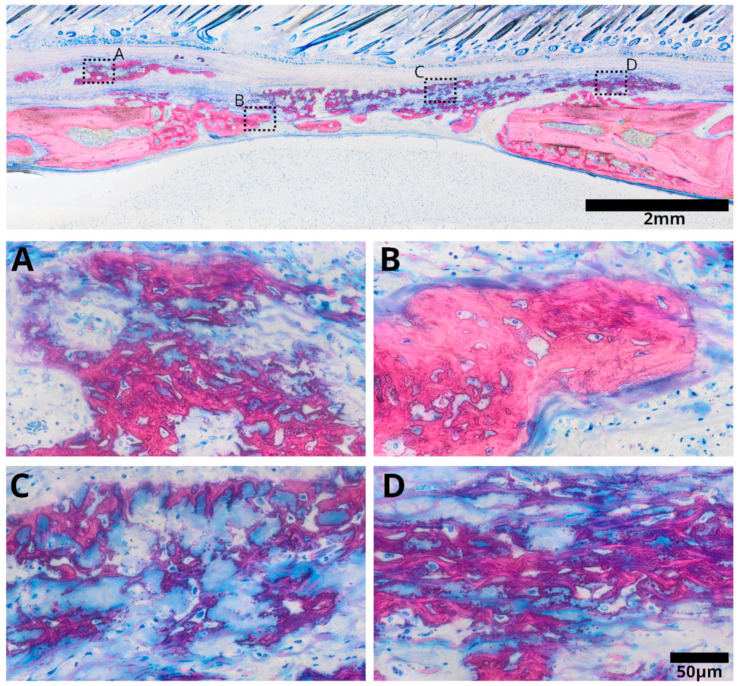
Overview image illustrating the collagen membrane immersed in rat platelet-rich fibrin (r-PRF) following a three-week healing period. Visible bone formation is apparent within the membrane (**A**), characterized by collagen fibers embedded into the new bone, indicative of a hybrid bone structure. In the vicinity of the inner defect near the brain (**B**), mature bone formation originating from the defect edges is observed, surrounded by collagen fibers derived from collagen matrix degradation. Remarkably, outside the defect, distinctive mineralization characteristics within the membrane are observed (**C**), where mineralized fibers from the collagen membrane appear to encapsulate surrounding cells, forming a reticular structure. Additionally, these mineralized collagen fibers form a network, contributing to hybrid bone formation (**D**).

Collagen membranes treated with r-PRF demonstrated strong bone formation, where most of the defect was filled with new bone (Figure 4). Distinctive regions were observed beneath and within the collagen membrane, showcasing varying characteristics. Near the edges of the membrane, mature bone formation was apparent, accompanied by residual collagen fibers. The observed bone pattern exhibited a lamellar structure, indicating maturity. Within the membrane, areas of bone formation displayed a distinct reticular structure, exhibiting areas with substantial bone deposition or mineralization alongside regions where only the fibers were mineralized. These mineralized fibers formed a hybrid bone structure. This structure entrapped cells, suggesting active cellular involvement in bone growth. This distinctive hybrid bone is indicative of bone growth within the collagen membrane treated with r-PRF.

### 3.4. Micro-CT Assessment of Defects Treated with Membranes Soaked in h-PRF and r-PRF

**Figure 5:**
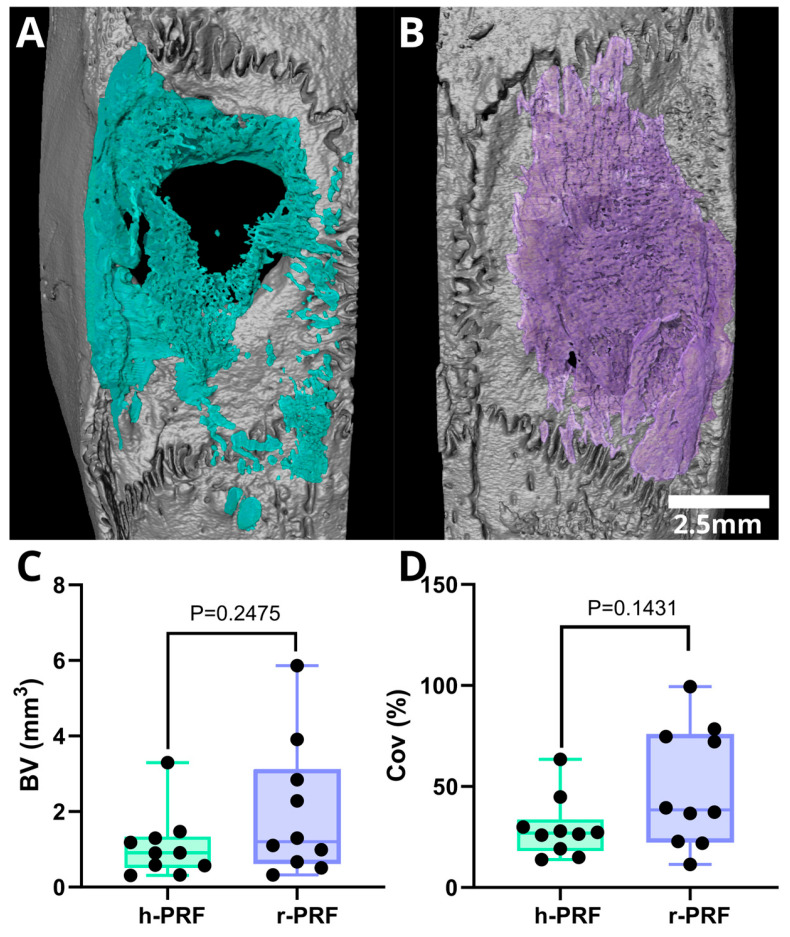
Three-dimensional renderings depicting defects treated with collagen membranes soaked in human PRF (h-PRF) (**A**) and rat PRF (r-PRF) (**B**). The parameters analyzed included bone volume (BV) and coverage (Cov%). Despite the seemingly higher bone volume and consequently better coverage in the r-PRF group, no statistically significant differences were observed between these groups (**C**,**D**).

**Figure 6:**
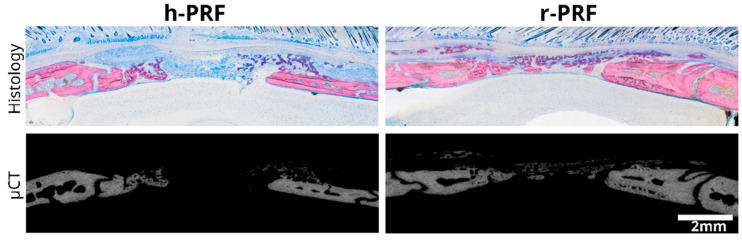
The micro-CT image validates the observations made in the histological analysis, showcasing a correlation between the two techniques. This combined display of histology and micro-CT enhances the comprehensive assessment of the tissue structure.

The median bone volume (BV) observed for the collagen membranes immersed in h-PRF and r-PRF was 0.9 mm^3^ (range: 0.3–3.3 mm^3^) and 1.2 mm^3^ (range: 0.3–5.9 mm^3^), respectively. Consequently, the median defect coverage was 26.9% (raning from 13.8–63.4%) and 38.4% (ranging from 11.5–99.4%) for h-PRF and r-PRF as depicted in Figure 5. The analysis further delved into the median bone volume fraction (BV/TV) which was found to be 2% (with a range of 0.5 to 7.2%) for h-PRF and 6.5% (range: 0.8–11.8%) for r-PRF, respectively. Trabecular thickness, another critical parameter, exhibited a median of 0.13 mm^3^ (range: 0.08–0.19 mm^3^) for h-PRF, while the median for the r-PRF-immersed membranes was 0.11 mm^3^ (range: 0.1–0.16 mm^3^). Moreover, it is imperative to underscore additional noteworthy characteristics of the micro-CT analysis, particularly when correlated with the histology sections, where only mineralized structures are visible (as illustrated in Figure 6).

## 4. Discussion

PRF research is designed to optimize human PRF based on in vitro studies; however, preclinical testing using human PRF may have drawbacks. Therefore, this study was driven by the overall question of whether the preparation of human PRF can be tested using the established model of rat calvaria defects—in particular, a variation of the model where the defect is covered by a collagen membrane [25,26,27,28,29,30,31]. This collagen membrane allowed for active and passive mineralization [28,29], which was ideal for studying the impact of its functionalization in the present study with human PRF and rat PRF. The main finding was that, compared to rat PRF, coating the collagen membrane with human PRF reduces new bone formation and passive mineralization within the spongy part of the biomaterial. This finding is based on a combination of histological evidence and a micro-CT analysis of the defect site and the adjacent area. The present observation is important because it reminds us of the limitations of using rodent models to study biomaterials functionalized with human PRF.

Our study supports previous research; for instance, rabbit PRF was tested in rabbits with a 3D bilayer collagen membrane reinforced with β-TCP, resulting in approximately 30% new bone formation after four weeks [33]. Also, when using rat PRF alone or in combination with a mineralized biomaterial, the rat calvaria defect models allowed bone formation of around 10% and 30%, respectively, after four weeks [23]. Another source of support comes from using a coagulated rat PRP in rat calvaria defects, resulting in approximately 6 mm^3^ of new bone after a four-week observation period [42]. One study used human PRF as a carrier for cells to treat an immunodeficient mouse calvaria defect, resulting in 2.5 mm^3^ of new bone in the defect [34]. Thus, no studies regarding heterologous PRF on bone regeneration in immunocompetent rodent models exist. However, this approach seems feasible in wound healing research. Care should be taken when interpreting the areas of mineralization as bone; the mineralized fibers, even when cells are occasionally entrapped in the newly formed matrix, are devoid of the typical osteoblast seams, and their staining is more intense than regular bone. Thus, the conclusions should not be restricted to bone regeneration but to the general mineralized matrix.

In wound healing, at least two studies have compared human platelet-rich plasma (PRP) and PRF with homologous preparations. For instance, human PRP showed more tissue retraction at the wound healing process’s beginning than rabbit PRP [35]. In dog skin punch defects, PRP gels of human and dog origin were equivalent in their ability to accelerate wound healing after 17 days [43]. There is thus a reason to follow up on the wound healing concepts to test heterologous preparations of PRF and translate this into a bone regeneration model such as the calvaria defect. In contrast to the two wound healing studies [35,43], we show that rat (but not human) PRF supports the osteoconductive properties of a collagen membrane when placed in a rat calvaria defect. The underlying reason remains unclear, but we know that collagen membranes functionalized with the conditioned medium of human PRF—thus, not containing the cells and the fibrin-rich matrix of the PRF membranes—result in around 50% defect coverage after four weeks [30]. Thus, mainly human cells and the fibrin-rich matrix hinder rat tissues from consolidating and forming new bone tissue.

Future research should focus on the hypothesis that plasminogen activators originating from rat cells are less capable of initiating the fibrinolytic process in a human fibrin-rich matrix, as already suggested by a study comparing the responsiveness of human recombinant tissue-type plasminogen activators between rats and humans [44]. Moreover, there have been attempts to compare the clotting properties of rat and human blood [45]. Support for this concept comes from showing that mice lacking plasminogen are incapable of bone regeneration, but, interestingly, mice lacking fibrinogen can do so [46]. Thus, fibrinolysis is more important than the formation of a fibrin-rich matrix in bone regeneration [46]. We must also consider the possibility that the cells in human PRF (actually the C-PRF rich in platelets and leucocytes [38,39,40]), provoke a local immune reaction that suppresses bone formation overall, which is typically observed at the site of chronic inflammatory osteolysis [47]. Even though the reason why human PRF reduces bone formation in a rat calvaria defect model remains to be determined, we can draw a conclusion on a descriptive level, namely that rat models are not ideal for studying the in vivo behavior of biomaterials functionalized with human PRF.

The present study has several limitations that should be acknowledged. Firstly, we were unable to investigate the molecular mechanisms underlying bone formation for each treatment, which could provide valuable insights into the mechanisms of action. Additionally, our study only included a single time point for observation, focusing on the early healing stages. While this allowed us to evaluate initial differences between the treatment groups, a longer-term follow-up would have provided a more comprehensive understanding of the treatment effects over time.

## 5. Conclusions

The rat calvaria defect is an accepted model to determine the possible impact of rat PRF on bone regeneration, but not necessarily that of PRF from human blood. In support of this claim, we show that bone regeneration in defects treated with Bio-Gide^®^ collagen membranes soaked with rat PRF is more pronounced than in membranes treated with human PRF, based on histology and micro-CT. These observations imply that caution is necessary when generalizing conclusions about the efficacy of human PRF in the rat calvaria defect model.

## Data Availability

The data sets generated during this study are available upon request to the corresponding author.

## Supplementary Materials

The following supporting information can be downloaded at https://www.mdpi.com/article/10.3390/bioengineering11050414/s1, Table S1. Post hoc analysis for BV/TV (%) comparing Empty defect with human and rat PRF. Table S2. Post hoc for coverage (%) comparing Empty defect with human and rat PRF.
