# Supplementary material for: Human versus Rat PRF on Collagen Membranes: A Pilot Study of Mineralization in Rat Calvaria Defect Model
Source: Bioengineering (Basel). 2024 Apr 23;11(5):414. doi: 10.3390/bioengineering11050414 (PMC11117948; doi:10.3390/bioengineering11050414)
Supplement: Supplementary file 1 [file bioengineering-11-00414-s001.zip › bioengineering-2889019-supplementary.pdf]

Supplementary Material

Table S1. Post hoc for BV/TV (%) comparing Empty defect with human and Rat PRF.

	Values	Sigma	Values
ED vs. h-PRF	0.02	0.00	<0.001
ED vs. r-PRF	-0.00	0.01	0.998

Table S2. Post hoc for Coverage (%) comparing Empty defect with human and Rat PRF.

	Values	Sigma	Values
ED vs. h-PRF	-0.04	0.00	<0.001
ED vs. r-PRF	-0.26	0.08	0.00373
